# Spatial Models With Inter-Tree Competition From Airborne Laser Scanning Improve Estimates of Genetic Variance

**DOI:** 10.3389/fpls.2020.596315

**Published:** 2021-01-07

**Authors:** David Pont, Heidi S. Dungey, Mari Suontama, Grahame T. Stovold

**Affiliations:** ^1^Forest Informatics, Scion, Rotorua, New Zealand; ^2^Forest Genetics, Scion, Rotorua, New Zealand; ^3^Tree Breeding, Skogforsk, Umeå, Sweden

**Keywords:** spatial analysis, tree competition, environment, tree phenotyping, airborne laser scanning, heritability, field trial

## Abstract

Phenotyping individual trees to quantify interactions among genotype, environment, and management practices is critical to the development of precision forestry and to maximize the opportunity of improved tree breeds. In this study we utilized airborne laser scanning (ALS) data to detect and characterize individual trees in order to generate tree-level phenotypes and tree-to-tree competition metrics. To examine our ability to account for environmental variation and its relative importance on individual-tree traits, we investigated the use of spatial models using ALS-derived competition metrics and conventional autoregressive spatial techniques. Models utilizing competition covariate terms were found to quantify previously unexplained phenotypic variation compared with standard models, substantially reducing residual variance and improving estimates of heritabilities for a set of operationally relevant traits. Models including terms for spatial autocorrelation and competition performed the best and were labelled ACE (autocorrelation-competition-error) models. The best ACE models provided statistically significant reductions in residuals ranging from −65.48% for tree height (*H*) to −21.03% for wood stiffness (*A*), and improvements in narrow sense heritabilities from 38.64% for *H* to 14.01% for *A*. Individual tree phenotyping using an ACE approach is therefore recommended for analyses of research trials where traits are susceptible to spatial effects.

## Introduction

The development of a precision approach to forestry can improve the efficiency and sustainability of managed forests. The aspiration is to utilize improved tree breeds, planted on the most suitable sites, and managed to optimize production, while minimizing costs and environmental impacts by targeted applications of inputs such as fertilizers (Dungey et al., 2018). A critical requirement for precision forestry is accurate and cost-effective methods to characterize individual trees (Tsaftaris et al., 2016; D’odorico et al., 2020). This capability could not only be utilized in trials to support research into improved breeds for tree growth and quality, but also in assessment of trees for inventory of forest stands at different stages of the production cycle. Traditional forest inventory and trial measurement rely on ground-based measurement of traits such as tree diameter, height, and volume. Such measurements are time consuming and error-prone. Remote sensing offers the potential for high throughput, accurate, and spatially explicit phenotyping, providing an essential foundation for precision management (Fahlgren et al., 2015). At this stage, airborne laser scanning has been most successfully adopted for forest inventory internationally, typically using area-based methods to characterize patches of the order of 0.04 ha in size (White et al., 2013; Maltamo et al., 2014). Alternative methods, identifying and delineating individual trees using ALS, have been developed and evaluated on New Zealand radiata pine stands (Pont, 2016).

Radiata pine (*Pinus radiata D. Don*) is the dominant tree species in the New Zealand forest estate comprising 90% of the planted area (Forest Owners Association, 2019). The New Zealand radiata pine forest estate comprises a monospecific, even aged, intensively managed forest crop. Considerable levels of variation in tree attributes remain, due to genetics, environment, and silviculture (Dungey et al., 2006). Forest tree breeding requires the evaluation of large numbers of trees and sufficient replication due to genetic variability, resulting in trials containing thousands of trees, and occupying several hectares (Dungey et al., 2009).

A standard approach to mitigate the effects of environmental variation in genetics trials is the use of spatial terms in analytical models (Dutkowski et al., 2006). A useful method is the separable first-order autoregressive model (AR1) in two dimensions (Cullis et al., 1998), utilizing inverse distance-weighted correlations across rows and columns in the trial, allowing for differing spatial correlations in the row and column directions. This successfully accounts for various forms of environmental effects in trial analyses (Cullis et al., 2014). Competition is a form of negative autocorrelation, where neighbors of a larger tree are more likely to be smaller, and vice versa (Griffith and Arbia, 2010). Autocorrelative methods such as AR1 models do not distinguish positive and negative autocorrelation and as a result the two effects are confounded (Griffith and Arbia, 2010). In order to accurately account for environmental variation, methods to quantify both positive (site) and negative (competition) autocorrelation are needed (Cappa and Cantet, 2008; Costa et al., 2013; Dong et al., 2020).

Tree traits of primary importance to breeders and forest managers include size, wood quality and disease susceptibility. Height, diameter at breast height (DBH) and total stem volume are widely used fundamental measures of tree size and productivity, able to be estimated from tree-based analyses of ALS (Lindberg et al., 2013; Dalponte et al., 2018). Dothistroma needle blight (*Dothistroma septosporum* (Dorog.) M. Morelet) is a foliar disease causing considerable productivity losses (Watt et al., 2011), and wood stiffness is an wood quality characteristic important for structural uses of timber (Carson et al., 2014). Individual tree crown metrics derived from the ALS were shown to correlate with these aforementioned tree size, disease, and wood quality traits, and to provide accurate estimates of genetic parameters such as heritabilities (Pont, 2016).

Competition metrics express the growth potential of a tree relative to nearby trees, generally considering the size and proximity of those trees (Pretzsch, 2010; Burkhart and Tomé, 2012). The inclusion of such competition metrics in analytical models was of interest to partition competition effects from general environmental effects. Spatially registered crown metrics from tree-based analysis of ALS quantifying tree size and locations are suitable for derivation of individual tree competition metrics. In a review of competition metrics for use with individual tree analysis of ALS (Suárez, 2010), distance weighted size ratios were utilized. Such ratios, initially used by Hegyi (1974), were expressed in terms of tree diameters:

$$\text{CI}=\sum_{\text{j}-1}^{\text{n}}\left(\frac{{\text{d}}_{\text{j}}/{\text{d}}_{\text{i}}}{{\text{L}}_{\text{ij}}}\right)$$

where, d_*i*_ = DBH of reference tree i, d_*j*_ = DBH of competitor tree j, L_*ij*_ = distance between reference tree i and competitor j.

It is hypothesized that spatial models including autoregressive and competition terms reduce unexplained variation compared to conventional models lacking spatial terms. The ability to more accurately quantify spatial effects reduces model residuals and thereby improves heritability estimates. The study was carried out in a genetics trial, providing the unique opportunity to work within an experimental design of known spacing and documented genetics. Environmental effects due to competition and site related variation were evident in the selected trial, providing the opportunity to examine models accounting for these effects in a controlled setting.

## Materials and Methods

### Genetics Trial Site

**FIGURE 1:**
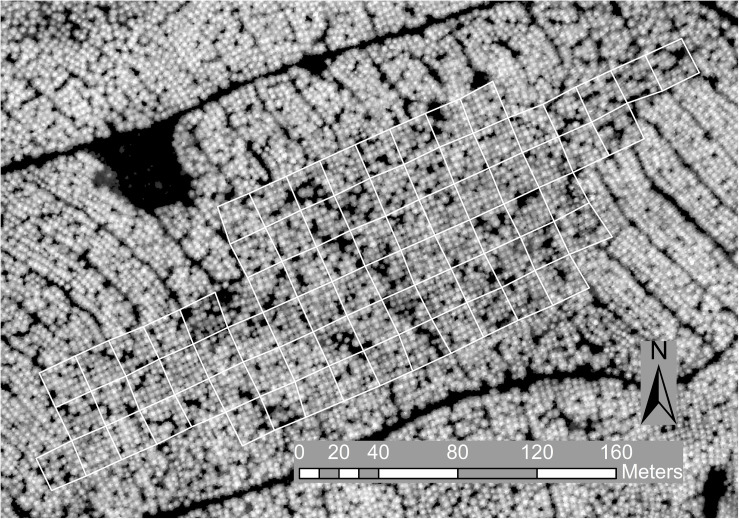
Trial layout with blocks outlined in white on the canopy height model image derived from the ALS data at 0.25 m resolution and used for tree detection.

The genetics trial BC 35-3 was established in 2007 by the Radiata Pine Breeding Company Ltd., in compartment 76 at Kaingaroa forest (38.53° S, 176.66° E) in the central North Island of New Zealand. The trial was designed to evaluate Dothistroma needle blight resistance for families in a breeding program and used an incomplete block design with single tree plots (Dungey et al., 2009). The trial site covered a total area of 2.8 ha and sloped gently (<5 degrees) to the southeast. The trial comprised 75 blocks, with 25 replicates and 3 incomplete blocks per replicate (Figure 1). Each block measured 19.2 × 19.2 m, with tree spacing on a uniform grid of 3.2 × 3.2 m. Blocks were established with 36 trees from different families in a 6 × 6 grid. Six control families were present in every block and the remaining 90 families were assigned across the three incomplete blocks for each replicate, with randomized spatial locations within the blocks and replicates. The surrounding stand was also established with radiata pine in 2007 at a density of 1000 stems ha^–1^ and thinned to 786 stems ha^–1^ in 2012.

**FIGURE 2:**
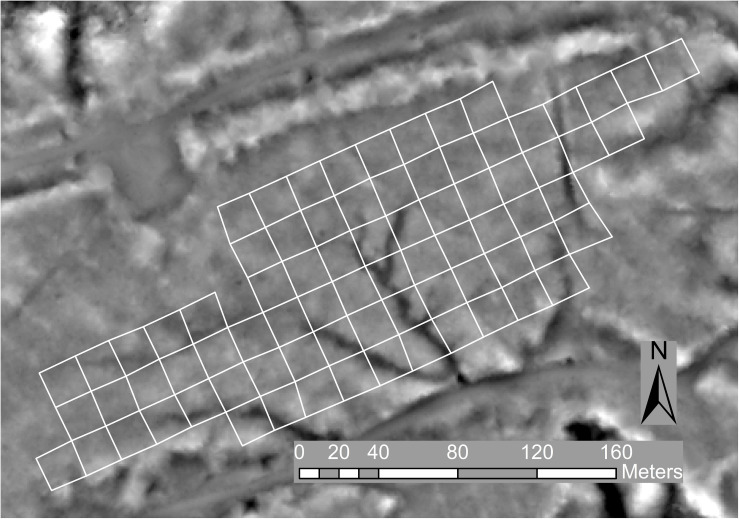
Trial layout with blocks outlined in white on an image of a digital terrain model (derived from the ALS data at 0.8 m resolution) shaded to indicate relative elevation and reveal a number of gullies evident as dark areas.

**FIGURE 3:**
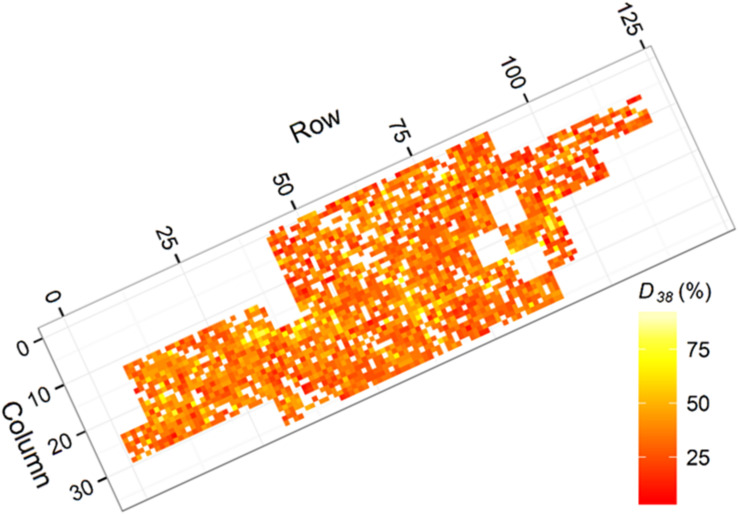
Heatmap presenting ground measured degree of Dothistroma infection (*D*_38_) for each tree (white indicates no data). Areas with elevated infection coincided with the gullies identified within the trial.

Initial inspection of the trial data showed evidence of spatial autocorrelation, with reduced height and diameter growth and increased levels of Dothistroma infection associated with small gullies within the trial (Figures 2, 3). Favorable conditions for Dothistroma are known to occur in gullies, where moist conditions persist, indicating the possibility of reduced tree growth associated with Dothistroma infection (Bulman et al., 2004). In addition to observable site effects, the trial had missing trees. Missing trees typically occur due to mortality, and in this trial dead, unhealthy, and highly malformed trees were also removed during the 2012 operational thinning of the surrounding stand. The resulting gaps in the trial grid created potential for competition effects (Fins et al., 1992).

### Field Measurement of Tree Traits

A ground-based assessment of a number of tree traits was carried out in July 2014 when the trial was aged 7 years, following standard tree breeding measurement methodologies (Jayawickrama, 2001). Measurements of tree diameter at breast height (*DBH*), height (*H*), and outerwood stress wave velocity (*A*), and degree of needle loss caused by Dothistroma needle blight (*D*_38_), were carried out. Tree *DBH* was measured using a fiberglass girth tape with diameter gradations at millimeter intervals (Friedrich Richter Messwerkzeuge GmbH & Co., Speichersdorf, Germany). Tree *H* was measured using a Vertex IV (Haglof, Sweden). Total stem volume (*V*) was estimated for each tree using the standard volume equation V182 (Goulding, 1995):

$$V=DBH^{a}{\left(\frac{H^{2}}{H-1.4}\right)}^{b}e^{c}$$

where *a* = 1.79068, *b* = 1.07473, *c* = −10.03201, and *e* is Euler’s number.

Outerwood stress wave velocity (*A*) was measured using a HITMAN ST300 (Fibre-gen Ltd., Christchurch, New Zealand) with the probes placed 1 m apart, avoiding knots and defects that could affect readings. The measure *A* is correlated with wood stiffness, an important engineering property for structural uses of timber (Carson et al., 2014). Degree of needle loss (*D*_38_) was assessed at age 38 months as a percentage of needles infected or lost, estimated visually in 5 percent increments (Van Der Pas et al., 1984). Individual tree *D*_38_ measurements are presented in Figure 3 where indication of increased infection in association with gullies crossing the trial (see Figure 2) was evident.

### Airborne Laser Scanning Data

The discrete return ALS data were acquired in early 2014 using an Optech Pegasus scanner with a pulse rate of 100 kHz, a maximum scan angle of ± 12°, a 25% swath overlap, and a 0.25 m footprint size. The data were georeferenced to the NZGD2000 NZTM coordinate system and all returns were classified as ground and above ground (using Terrascan TerraSolid software). The average point density of the point cloud over the trial area was 17 total returns per m^2^ and 7 last returns per m^2^.

### Crown Metrics

A canopy height model (CHM) with 0.25 m resolution was extracted from ALS data collected over the trial and image defects referred to as pits were removed using the standard image processing method *closing* (Ronse and Heijmans, 1991; Andersen et al., 2006). Individual trees were detected and crown boundaries determined on the CHM image using the calibrated ITC method (Pont et al., 2015). The method uses watershed segmentation with operator calibration to determine the level of image smoothing and has been shown to provide tree detection accuracy of 95% for New Zealand radiata pine across a range of stand densities and crown sizes. Detected trees were matched to ground trees with an automated least squares approach (Hauglin et al., 2014) and unmatched trees used to identify and manually correct segmentation errors.

Watershed segmentation resulted in growing space polygons, one per detected tree, which completely tiled the image, each including a tree crown and a portion of any adjacent gap between trees. Tree crown boundaries were then delineated within each growing space polygon to exclude any gap area. For tree crowns with no adjacent gaps, growing space and crown polygons were identical. The CHM image gray values within each crown represent heights above ground. Growing space boundaries, crown boundaries, and crown elevation values were used to derive a total of nine crown size metrics (Table 1) correlated with the traits of interest (Pont, 2016). Crown volumes (*CV*_*F*_ and *CV*_*P*_) quantified three-dimensional crown size, while surface areas from projected polygon outlines (*GA*_*P*_ and *CA*_*P*_) and from surface areas of three-dimensional crown surfaces (*CS*_*C*_ and *CS*_*T*_) provided two-dimensional measures of crown sizes. Crown length and radius (*CL* and *CR*) provided one-dimensional measures and the ratio of crown and growing space areas (*A*_*GC*_) provided a dimensionless measure of crown size. These crown size metrics were then used in competition metrics described subsequently.

**TABLE 1:** Individual tree crown metrics used in candidate competition metrics with most highly correlated tree size trait and Pearson’s *r*.

Abbreviation	Description	Units	Trait *r*
$CR=\sqrt{\frac{CA_{p}}{\pi }}$	Crown radius derived from crown area (*CA*_*p*_).	m	*DBH* 0.679
*CL*	Crown length, difference between crown highest point and average height of crown boundary points.	m	*H* 0.476
*GA*_*P*_	Two-dimensional ground area of crown growing space from watershed segmentation polygon.	m^2^	*DBH* 0.518
*CA*_*P*_	Two-dimensional ground area of crown determined from crown boundary polygon.	m^2^	*V* 0.574
*A*_*CG*_	Ratio of crown and growing space areas (*CA_*P*_/GA_*P*_*).	−	*H* 0.297
		m^2^	*V* 0.598
*CS*_*T*_	Surface area of triangulated crown CHM heights.	m^2^	*V* 0.574
*CV*_*F*_	The volume between the crown upper surface and the ground (Chen et al., 2007).	m^3^	*V* 0.844
*CV*_*P*_	The volume between the crown upper surface and the base of the crown.	m^3^	*DBH* 0.541

### Competition Metrics

Rouvinen and Kuuluvainen (1997) presented a set of competition metrics, *CI*10, *CI*11, and *CI*12, derived from the original by Hegyi (1974). Those competition metrics were evaluated by Suárez (2010) using ground measured *DBH* and using *DBH* estimated from LiDAR CHM crown metrics. Those competition models were generalized in our study to utilize the crown metrics (see Table 1) derived from the ALS CHM as:

$$\begin{array}{c} \text{CIA}=\sum_{\text{j}-1}^{\text{n}}\left(\frac{{\text{c}}_{\text{j}}/{\text{c}}_{\text{i}}}{{\text{L}}_{\text{ij}}}\right)\\ \text{CIB}=\sum_{\text{j}-1}^{\text{n}}\left({\frac{{\text{c}}_{\text{j}}/{\text{c}}_{\text{i}}}{{\text{L}}_{\text{ij}}}}^{2}\right)\\ \text{CIC}=\sum_{\text{j}-1}^{\text{n}}\left(\frac{{\left({\text{c}}_{\text{j}}/{\text{c}}_{\text{i}}\right)}^{2}}{{\text{L}}_{\text{ij}}}\right) \end{array}$$

where *c*_*i*_ = crown metric for reference tree i, *c*_*j*_ = crown metric for competitor tree j, *L*_*ij*_ = distance between reference tree i and competitor j.

Two methods were used for determining the neighboring trees included in the calculation of the competition metrics described above. In the area method (*N*_*A*_), all trees within a fixed radius were included, an approach used in a number of previous studies (Hegyi, 1974; Pukkala et al., 1994; Rouvinen and Kuuluvainen, 1997; Suárez, 2010). The grid spacing in the trial was used to estimate a radius of 8.273 m to include an average of twenty trees surrounding the central tree. In the boundary method (*N*_*B*_), only trees sharing a segment boundary (as delineated on the CHM) with the target tree were included (Suárez, 2010). Processing of the CHM included a 50 m buffer around the trial and competition metrics using both neighborhood methods included trees surrounding the trial, a distinction with competition metrics which often only account for trees measured within plots or trials (Dutkowski et al., 2006). The use of 9 crown metrics (Table 1) and 3 model formulations (CIA, CIB, CIC) gave 27 competition metrics. Use of the two neighborhood methods (*N*_*A*_ and *N*_*B*_) with the 27 metrics gave a total of 54 competition metrics for evaluation.

### Spatial Models

The following general individual tree linear mixed model was used as the basis of all spatial models:

y=Xb+Zu+e

where ***y*** is a vector of individual tree observations of a specific trait (*H*, *DBH*, *V*, *D*_38_ or *A*), ***b*** is a vector of fixed effects, ***u*** is a vector of random effects, and ***e*** is a vector of random residuals. The terms ***X*** and ***Z*** correspond to design matrices relating the observations in ***y*** to the fixed and random effects in ***b*** and ***u***, respectively (Dungey et al., 2012).

We fitted a model without spatial terms, as routinely used in estimating variance components and genetic parameters including narrow sense heritabilities, referred to as Base model (B) (Dutkowski et al., 2006). Fixed effects in vector ***b*** included the overall mean and a factor with two levels to account for the effects of control versus non-control material. Random terms in vector ***u*** included the additive genetic effects of individual genotypes for pedigreed material, the effects of replicates and the effects of incomplete blocks within replicates. We also fitted a standard spatial model using an auto-regressive order 1 random term, commonly referred to as AR1xAR1, and abbreviated to AR1 hereafter (Dutkowski et al., 2006). We referred to this standard spatial model as Base plus AR1 (BA). In this model vector ***e*** was partitioned into spatially correlated (**ξ**) and uncorrelated (**η**) residuals and replicate was removed as a fixed effect. The spatially correlated error **ξ**, was modeled by using a first-order separable autoregressive process in the row and column directions (Gilmour et al., 1997; Costa et al., 2001; and Dutkowski et al., 2002).

Competition metrics were then introduced as covariates to those models, referred to as Base plus Competition (BC) and Base plus AR1 plus Competition (BAC). Covariate values were standardized by subtracting the mean and dividing by the standard deviation (Butler et al., 2009). Control and the overall mean appeared as the only fixed terms, and the additive genetic effects of individual trees appeared as a random term, in all models. Trees were assigned to grid rows and columns, and missing values added to ensure a complete grid for AR1 models. A nugget effect (referred to as units) was added to models having an AR1 spatial term, as this has been shown to be significant in several studies (Suontama et al., 2015). Incomplete block by replicate was tried as a random term in all models, but it became non-significant when an AR1 spatial term was added, so it was dropped from those models (Dungey et al., 2012). The set of models evaluated are summarized in Table 2.

**TABLE 2:** Models fitted for each trait to compare improvements from random terms to account for spatial autocorrelation (AR1) and addition of competition covariates derived from analyses of the ALS tree crown metrics.

Model	Abbreviation	Fixed terms	Random terms
Base	B	control	replicate + iblock:rep + pedigree
Base + AR1	BA	control	pedigree + units + AR1
Base + Competition	BC	control	replicate + iblock:rep + pedigree + competition
Base + AR1 + Competition	BAC	control	pedigree + units + AR1 + competition

The B and BA models were fitted to each of the 5 traits (*H*, *DBH*, *V*, *D*_38_, *A*), requiring 10 model runs. The BC and BAC models were fitted for the 5 traits by 54 competition metrics, requiring 270 model runs. The total number of model runs being 280.

### Model Evaluation

Models can be compared with log-likelihood (LL) ratio tests if they have the same fixed effects, and if one model has a subset of the random effects in the other model (nested models) (Isik et al., 2017). Models with the same fixed effects that are not nested can be compared with information criteria, Akaike’s (AIC) or Schwarz’s Bayesian Information Criteria (BIC). In our study we evaluated four nested models with the same fixed effects (control only), presented in Table 2. We evaluated models under the premise that all models including some spatial component were alternative approaches to accounting for spatial variation compared to the base model. If *LL*_*M*_ and *LL*_*B*_ are the REML log-likelihoods for a test and base models, respectively, the test statistic (*D*) is given by:

$$D=2\left(LL_{M}-LL_{B}\right)$$

where Akaike Information Criteria (AIC) and Bayesian Information Criteria (BIC) used to rank models (Dutkowski et al., 2006) are derived as follows:

$$\begin{array}{l} AIC=-2LL_{Ri}+2t_{i}\\ BIC=-2LL_{Ri}+2t_{i}logv \end{array}$$

where *LL* is the log-likelihood of the model, *t*_*i*_ is the number of variance parameters in model *i*, and ν = *n − p* is the residual degrees of freedom.

Narrow sense heritabilities (*h*^2^) were estimated for each model fit, using the additive genetic variance as a ratio of the phenotypic variance, expressed as the sum of the additive and residual variances:

$$h^{2}=\frac{Var_{A}}{Var_{A}+Var_{E}}$$

where *Var*_*A*_ is the additive genetic variance and *Var*_*E*_ is the residual variance.

In the case of models with an AR1 term, the residual is represented by the units component (Dutkowski et al., 2002). Models where competition covariates terms were fitted for each trait (BC, and BAC) were ranked by LL and the best model selected. All models tested were ranked by LL (higher being better), and then associated *h*^2^ and residual variance components were examined. Two metrics were derived to express the improvements of spatial models compared to the base model, the change in *h*^2^, and in residual (ϵ), compared to the standard (base) model, multiplied by 100 to be expressed as percentages:

$$\begin{array}{c} \Delta {\text{h}}^{2}=\frac{{\text{h}}_{\text{M}}^{2}-{\text{h}}_{\text{B}}^{2}}{{\text{h}}_{\text{B}}^{2}}\\ \Delta \epsilon =\frac{\epsilon_{\text{M}}-\epsilon_{\text{B}}}{\epsilon_{\text{B}}} \end{array}$$

where the subscripts *M* and *B* represent the spatial and base models, respectively.

## Results

Spatial models provided statistically significant improvements in LL over a base model for all traits (*H*, *D*, *V*, *D*_38_, and *A*), the ranking order in Table 3 was BAC, BC, BA. Spatial BAC models resulted in reductions in residual ranging from −65.48% for *H* to −21.03% for *A*, and improvements in *h*^2^ from 38.64% for *H* to 14.10% for *A*. Results confirmed prior research that auto-regressive order 1 (AR1) models are able to account for spatial effects from a wide variety of sources (Isik et al., 2017). Results also showed the utilization of a competition covariate in combination with an AR1 term explained additional variation, substantially reducing the residual and improving *h*^2^ for all traits except *D*_38_. The tree size traits *DBH*, *H*, and *V* benefited the most from competition covariates, and the greatest improvements in residual and *h*^2^ relative to a BA model were −55% and 29%, respectively from a BAC model for *DBH*.

**TABLE 3:** ASReml model fit statistics Log-likelihood (LL) and test statistic (*D*) and results heritability (*h*^2^) with its standard error (SE), by trait and model, ordered by decreasing Log-likelihood (LL) within trait and traits are ordered by decreasing improvement in residual (Δε*%*) from the best model, improvement in heritability (Δ*h*^2^%) is also shown.

Trait	Model	LL	*D*	*h*^2^	SE	Δ *h*^2^%	Δε%
*H*	BAC	−1291	<0.001	0.4117	0.0950	38.64	−65.48
	BC	−1381	<0.001	0.3011	0.0679	1.39	−32.90
	BA	−1758	<0.001	0.3425	0.0770	15.34	−24.87
	B	−1800	<0.001	0.2969	0.0660	−	−
*DBH*	BAC	−8406	<0.001	0.3753	0.0899	33.55	−63.65
	BC	−8440	<0.001	0.2954	0.0657	5.13	−38.90
	BA	−8930	<0.001	0.2950	0.0671	4.99	−8.58
	B	−8948	<0.001	0.2810	0.0636	−	−
*V*	BAC	5757	<0.001	0.3642	0.0856	22.47	−56.26
	BC	5742	<0.001	0.2980	0.0649	0.21	−31.89
	BA	5347	<0.001	0.3140	0.0678	5.60	−8.87
	B	5334	<0.001	0.2973	0.0640	−	−
*D*_38_	BAC	−6024	<0.001	0.4912	0.0931	34.88	−50.31
	BC	−6096	<0.001	0.3546	0.0705	−2.63	−14.43
	BA	−6210	<0.001	0.4926	0.0922	35.28	−40.18
	B	−6276	<0.001	0.3642	0.0713	−	−
*A*	BAC	1052	<0.001	0.4862	0.1101	14.10	−21.03
	BC	1047	<0.001	0.4221	0.0764	−0.93	−0.30
	BA	1038	0.002	0.4531	0.0819	6.34	−9.63
	B	1033	<0.001	0.4261	0.0772	−	−

The use of competition covariates in spatial models without an AR1 term (BC models) resulted in statistically significant improvements in LL, and reductions in residuals. However, the BC models did not generally result in increased *h*^2^, the best improvements being 5.13% and −2.63% for *D* and *D*_38_, respectively. The sign and strength of Pearson’s correlation coefficients (*r*) were examined to elucidate the relationships between crown metrics and traits (see Table 4).

**TABLE 4:** Best performing (least Log-Likelihood) crown metrics in BAC models compared to the BA model by trait.

Trait	Model	Crown metric	N/CI	*r*
*H*	BAC	*CV*_*F*_	*N*_*B*_ *CIA*	−0.5436
	BA			
*DBH*	BAC	*CV*_*F*_	*N*_*B*_ *CIA*	−0.5990
	BA			
*V*	BAC	*CV*_*F*_	*N*_*B*_ *CIA*	−0.5423
	BA			
*D*_38_	BAC	*CV*_*F*_	*N*_*A*_ *CIA*	0.3971
	BA			
*A*	BAC	*A*_*CG*_	*N*_*A*_ *CIA*	0.1328
	BA			

*The competition crown metric, neighborhood definition (N) and competition index (CI) used are shown along with Pearson’s correlation coefficient (*r*) for the trait and crown metric.*

The crown metric *CV*_*F*_ (see Table 1) appeared in the competition metric for the top performing BAC models for all traits except *A*. Models with competition metrics based on the *CV*_*F*_ crown metric were consistently ranked highest, but only marginally higher than models with the *CR* and *CA*_*P*_ crown metrics (results not shown). Those three metrics represented crown size in terms of volume, radius, and projected area, respectively. Moderate to strong correlations were observed for competition metrics with tree size traits (*H*, *DBH*, and *V*, with *r* from −0.54 to −0.60) and with disease expression (*D*_38_, *r* = 0.40). Results associated higher competition metrics with reduced tree size, and increased disease levels.

Competition metrics based on distance-weighted crown size metrics (*CIA*, *CIB*, *CIC*) were derived using area (*N*_*A*_) and boundary (*N*_*B*_) neighborhoods. The *CIA* formulation, representing a linear distance weighting of crown sizes, appeared in the best BAC models for all traits (Table 4) and the boundary neighbourhood definition (*N*_*B*_) performed best for the tree size traits (*H*, *DBH*, and *V)*. The exponentially weighted derivations of competition index (*CIB* and *CIC*) were inferior.

## Discussion

### The Autocorrelation-Competition-Error Approach

Results from our study have confirmed the recognized general utility of AR1 spatial models with a spatially independent units term to take account of spatial environmental variation (Costa et al., 2001; Dutkowski et al., 2006). However, spatial autocorrelation can be positive, representing various site effects such as temperature and aspect, and negative, representing competition effects. Competition effects can result in the mutual masking of these forms of autocorrelation (Griffith and Arbia, 2010). Where negative autocorrelation is left unaccounted for, it can even result in insignificant spatial autocorrelation test statistics. The inclusion of competition effects to reduce residual variation was described in a theoretical approach, focused on competition of genetic origin, and showed failure to account for competition resulted in biased model estimates and increased residual variation (Costa et al., 2013). Our study was a practical demonstration of the presence of both positive (site) and negative (competition) autocorrelation, and the best models combined AR1 and competition terms to explain these respective environmental sources of variation. Models having an explicit competition term and a generic term to account for positive autocorrelation are referred to as ACE (autocorrelation-competition-error) models. Results confirmed our hypothesis that models including autoregressive and competition terms reduce residuals and improve estimates of heritabilities compared to conventional models without spatial terms.

The success of ACE models was attributed to three key features. Firstly, it was critical to separate negative and positive autocorrelation, representing variation due to competition and other environmental effects, respectively, which were otherwise confounded. Secondly, the inverse-distance weighted competition metrics employed successfully accounted for competition. Thirdly, we observed there are potentially numerous positively auto-correlated environmental effects on tree growth besides competition, which were robustly accounted for by an autoregressive model component. We suggest ACE models as a useful approach to analyses of tree and plant growth due to the ubiquity of both competition and site effects. Thus, we propose that it will be beneficial for modelers to test for, and quantify, those effects with ACE models, when possible, as a useful evolution of the currently recommended practice of applying AR1 models.

The competition and crown metrics utilized were parsimonious, easy to derive, and to interpret, which will aid application of ACE models. The competition metrics were of a widely recognized and applied form, utilizing positions and relative sizes of neighboring trees (Maleki et al., 2015). The crown metrics represented fundamental crown morphological features, and were derived from analysis of the CHM, a model of the upper surface of the canopy derived from the three-dimensional point cloud created by laser scanning (Pont et al., 2013). This is referred to as a raster-based approach, and is contrasted with point- and voxel-based methods which derive numerous measures from the three-dimensional point cloud, a majority of which are statistical measures of point dispersion (Zhen et al., 2016). The latter methods can yield large numbers of metrics, but we note that studies evaluating such metrics have typically found that a more limited set of crown morphological metrics, representing crown features such as diameter, length, area, and volume, were typically among the most useful variables in models estimating tree attributes (Vauhkonen et al., 2016). Raster based metrics derived using the methods described in this study permit the use of widely available point cloud data, either from ALS or photogrammetric methods (Krause et al., 2019), making the methods flexible and amenable to operational uses in forestry. Future research could compare the efficacy of ALS and photogrammetric data sources with ACE models.

### Generality of ACE Models

An autoregressive model relates a characteristic of a target tree to the same characteristic of its neighbors, reflecting Tobler’s first law of geography (Tobler, 1970). Autoregressive models make no attempt to explain effects or cause, and therein lies their power. We postulate that an AR1 term can effectively account for spatial effects from multiple sources and scales, particularly once a separate term for competition is included. As a hypothetical example of scale independence, consider a group of neighboring trees where height growth is being positively affected by soil fertility at a short scale, and negatively affected by temperature at a much larger scale. There could also be additional unknown influences on growth, all operating at different scales. An autoregressive model is agnostic to the factors or scales at play and utilizes the integrated result of all such effects on neighboring trees as a robust proxy for the effects on the target tree. The ACE approach could therefore robustly account for environmental variation of unknown sources and scales with an AR1 term, and is also amenable to the addition of terms representing explicit environmental effects as they are elucidated in future research.

### Traits Have Distinct Environmental Responses

The ACE approach was shown to be beneficial for a range of traits, but it was also apparent that the relative amounts of variation due to genotype, competition, and site were distinct by trait. Tree *DBH* and *V* apparently had strong overall spatial variation, predominantly due to competition, while *H* also exhibited strong spatial variation due to nearly equal amounts of competition and site effects. The weaker competition effect noted for Dothistroma infection agreed with a predominant site effect due to the localized spread of the disease, reliant on a water-borne transmission, and re-infection of trees from fallen needles (Bulman et al., 2013) and the known tendency for the disease to occur in gullies due to increased moisture and reduced air movement (Bulman et al., 2004). The smaller, but evident, competition effect for *D*_38_ could reflect reduced tree growth resulting from the disease, or increased infection of smaller trees.

### Applications

There are several important operational applications for tree level phenotyping which could be supported by the ACE modeling approach, ranging from trial, stand, and forest levels, for breeding, research, and management objectives. The use of ALS data provides accurate tree locations and sizes for use in competition metrics. It should be noted the modeling approach is even applicable to conventional ground measurement of trials, where remote sensed crown metrics are not available. In that case competition metrics could be derived from ground measured tree size, or other traits. The use of ACE models including known genetics in analysis of a genetics trial was shown to substantially reduce model residuals and improve heritabilities, potentially improving breeding values, tree selection, and future breeds. The benefits of improved tree breed selection for forest sites are accentuated in the context of climate change (Dungey et al., 2018; D’odorico et al., 2020; De Los Campos et al., 2020). The ACE method is also advocated for use in general research trial analyses to improve accuracy and precision of results by accounting for environmental variation. In this study ACE models were applied to tree size (*H*, *DBH*, *V*), disease expression (*D*_38_) and wood quality (*A*), representing a set of traits of primary importance to tree breeders and forest managers.

## Conclusion

Crown metrics utilized in competition metrics substantially reduced residual variation and improved heritabilities for a range of operationally relevant traits. Tree height, DBH, volume, Dothistroma infection and stiffness exhibited significant variation attributable to spatial environmental effects. Analyses showed that traits exhibited distinct combinations of genotypic, competition, and site related variation, which needs be considered when modeling. The crown metrics and competition metrics identified in this study were parsimonious, effective, and warrant further investigation.

Analyses of results lead to the proposal of ACE models as a robust and effective approach to account for environmental variation in tree traits. Those models comprise an explicit competition term accounting for negative autocorrelation and a generic spatial term to account for positively autocorrelated site effects. Inclusion of a competition term, which we derived from individual tree crown metrics, was observed to be critical to improving the effectiveness of spatial modeling for environmental effects, avoiding the confounding of negative and positive autocorrelation.

The ACE approach is recommended for wider evaluation in tree and plant growth analyses, particularly for size and disease attributes. The analysis of remotely sensed data using ACE models will be developed and evaluated in future studies to determine utility in improving accuracy of trial analyses, identification of superior trees, tree growth research, and precision forest management.

## Data Availability Statement

The raw data supporting the conclusions of this article will be made available by the authors, without undue reservation.

## Author Contributions

DP developed the crown and competition metrics, carried out model fitting, and wrote the core of the manuscript. DP, HD, and MS contributed to development of modeling approach. GS carried out field ground truth data collection. All authors participated in the writing and review of the manuscript, contributed to the article, and approved the submitted version.

## Conflict of Interest

The authors declare that the research was conducted in the absence of any commercial or financial relationships that could be construed as a potential conflict of interest.

## References

[B1] Andersen H. E. Reutebuch S. E. Mcgaughey R. J. (2006). A rigorous assessment of tree height measurements obtained using airborne LiDAR and conventional field methods. *Can. J. Remote Sens.* 32 355–366. 10.5589/m06-030 17060990

[B2] Bulman L. S. Dick M. A. Ganley R. J. Mcdougal R. L. Schwelm A. Bradshaw R. E. (2013). “Dothistroma needle blight,” in *Infectious Forest Diseases*, eds Gonthier P. Nicolotti G. (Wallingford: CAB International).

[B3] Bulman L. S. Gadgil P. D. Kershaw D. J. Ray J. W. (2004). *Assessment and control of Dothistroma needle blight Forest Research Bulletin*, (New Zealand: Forest Research).

[B4] Burkhart H. E. Tomé M. (2012). “Indices of individual-tree competition,” in *Modeling Forest Trees and Stands*, eds Burkhart H. E. Tomé M. (Dordrecht: Springer Netherlands), 201–232. 10.1007/978-90-481-3170-9_9

[B5] Butler D. G. Cullis B. R. Gilmour A. R. Gogel B. J. (2009). *ASReml**-R reference manual.* The State of Queensland: The Department of Primary Industries and Fisheries.

[B6] Cappa E. P. Cantet R. J. C. (2008). Direct and competition additive effects in tree breeding: Bayesian estimation from an individual tree mixed model. *Silvae Genetica* 57 45–56. 10.1515/sg-2008-0008

[B7] Carson S. D. Cown D. J. Mckinley R. B. Moore J. R. (2014). Effects of site, silviculture and seedlot on wood density and estimated wood stiffness in radiata pine at mid-rotation. *N. Zealand J. Forestry Sci.* 44 1–12. 10.1186/s40490-014-0026-3

[B8] Chen Q. Gong P. Baldocchi D. Tian Y. Q. (2007). Estimating basal area and stem volume for individual trees from LiDAR data. *Photogram. Eng. Remote Sens.* 73 1355–1365. 10.14358/PERS.73.12.1355

[B9] Costa E. Silva J. Kerr R. J. (2013). Accounting for competition in genetic analysis, with particular emphasis on forest genetic trials. *Tree Genet. Genom.* 9 1–17. 10.1007/s11295-012-0521-8

[B10] Costa E. Silva J. Dutkowski G. W. Gilmour A. R. (2001). Analysis of early tree height in forest genetic trials is enhanced by including a spatially correlated residual. *Can. J. Forest Res.* 31 1887–1893. 10.1139/x01-123

[B11] Cullis B. R. Jefferson P. Thompson R. Smith A. B. (2014). Factor analytic and reduced animal models for the investigation of additive genotype-by-environment interaction in outcrossing plant species with application to a *Pinus radiata* breeding programme. *Theor. Appl. Genet.* 127 2193–2210. 10.1007/s00122-014-2373-0 25145447

[B12] Cullis B. Gogel B. Verbyla A. Thompson R. (1998). Spatial analysis of multi-environment early generation variety trials. *Biometrics* 54 1–18. 10.2307/2533991

[B13] Dalponte M. Frizzera L. Ørka H. O. Gobakken T. Næsset E. Gianelle D. (2018). Predicting stem diameters and aboveground biomass of individual trees using remote sensing data. *Ecol. Indicat.* 85 367–376. 10.1016/j.ecolind.2017.10.066

[B14] De Los Campos G. Perez-Rodriguez P. Bogard M. Gouache D. Crossa J. (2020). A data-driven simulation platform to predict cultivars’ performances under uncertain weather conditions. *Nat. Commun.* 11:4876. 10.1038/s41467-020-18480-y 32978378 PMC7519145

[B15] D’odorico P. Besik A. Wong C. Y. S. Isabel N. Ensminger I. (2020). High-throughput drone-based remote sensing reliably tracks phenology in thousands of conifer seedlings. *N. Phytolog.* 226 1667–1681. 10.1111/nph.16488 32157698

[B16] Dong L. Xie Y. Wu H. X. Sun X. (2020). *Spatial and competition models increase the progeny testing efficiency of Japanese larch^∗^.* 10.1139/cjfr-2020-0007

[B17] Dungey H. S. Brawner J. T. Burger F. Carson M. Henson M. Jefferson P. (2009). A new breeding strategy for *Pinus radiata* in New Zealand and New South Wales. *Silvae Genetica* 58 28–38. 10.1515/sg-2009-0004

[B18] Dungey H. S. Dash J. P. Pont D. Clinton P. W. Watt M. S. Telfer E. J. (2018). Phenotyping Whole Forests Will Help to Track Genetic Performance. *Trends Plant Sci.* 23 854–864. 10.1016/j.tplants.2018.08.005 30217472

[B19] Dungey H. S. Matheson A. C. Kain D. Evans R. (2006). Genetics of wood stiffness and its component traits in *Pinus radiata*. *Can. J. Forest Res.* 36 1165–1178. 10.1139/x06-014

[B20] Dungey H. S. Russell J. H. Costa E. Silva J. Low C. B. Miller M. A. (2012). The effectiveness of cloning for the genetic improvement of Mexican white cypress *Cupressus lusitanica (Mill.)*. *Tree Genet. Genom.* 9 443–453. 10.1007/s11295-012-0565-9

[B21] Dutkowski G. W. Costa E. Silva J. Gilmour A. R. Wellendorf H. Aguiar A. (2006). Spatial analysis enhances modelling of a wide variety of traits in forest genetic trials. *Can. J. Forest Res.* 36 1851–1870. 10.1139/x06-059

[B22] Dutkowski G. W. Silva J. C. E. Gilmour A. R. Lopez G. A. (2002). Spatial analysis methods for forest genetic trials. *Can. J. Forest Res.* 32 2201–2214. 10.1139/x02-111

[B23] Fahlgren N. Gehan M. A. Baxter I. (2015). Lights, camera, action: High-throughput plant phenotyping is ready for a close-up. *Curr. Opinion Plant Biol.* 24 93–99. 10.1016/j.pbi.2015.02.006 25733069

[B24] Fins L. Friedman S. T. Brotschol J. V. eds (1992). *Handbook of quantitative forest genetics.* Dordrecht: Springer Science & Business Media 10.1007/978-94-015-7987-2

[B25] Forest Owners Association. (2019). *New Zealand Plantation Forest Industry Facts and figures.* New Zealand: Forest Owners Association.

[B26] Gilmour A. R. Cullis B. R. Verbyla A. P. (1997). Accounting for natural and extraneous variation in the analysis of field experiments. *J. Agr. Biol. Environ. Stat.* 2, 269–293. 10.2307/1400446

[B27] Goulding C. J. (1995). “Individual tree volume, taper, bark, and breakage equations,” in *Forestry handbook*, ed Edn, ed. Hammond D. (New Zealand: New Zealand Institute of Foresters (Inc.)), 115–116.

[B28] Griffith D. A. Arbia G. (2010). Detecting negative spatial autocorrelation in georeferenced random variables. *Int. J. Geograp. Inform. Sci.* 24 417–437. 10.1080/13658810902832591

[B29] Hauglin M. Gobakken T. Astrup R. Ene L. Næsset E. (2014). Estimating single-tree crown biomass of norway spruce by airborne laser scanning: A comparison of methods with and without the use of terrestrial laser scanning to obtain the ground reference data. *Forests* 5 384–403. 10.3390/f5030384

[B30] Hegyi F. (1974). “A simulation model for managing jackpine stands Growth models for tree and stand simulation,” in *Proceedings of the IUFRO Meeting*, Sweden: Royal College of Forestry 74–90.

[B31] Isik F. Holland J. Maltecca C. Whetten R. (2017). *Genetic data analysis for plant and animal breeding.* New York, NY: Springer International Publishing 10.1007/978-3-319-55177-7

[B32] Jayawickrama K. J. S. (2001). Genetic parameter estimates for radiata pine in New Zealand and New South Wales: A synthesis of results. *Silvae Genetica* 50 45–53.

[B33] Krause S. Sanders T. G. M. Mund J.-P. Greve K. (2019). UAV-Based Photogrammetric Tree Height Measurement for Intensive Forest Monitoring. *Remote Sens.* 11:758 10.3390/rs11070758

[B34] Lindberg E. Holmgren J. Olofsson K. Wallerman J. Olsson H. (2013). Estimation of tree lists from airborne laser scanning using tree model clustering and k-MSN imputation. *Remote Sens.* 5 1932–1955. 10.3390/rs5041932

[B35] Maleki K. Kiviste A. Korjus H. (2015). Analysis of individual tree competition on diameter growth of Silver Birch in Estonia. *Forest Syst.* 24:e023 10.5424/fs/2015242-05742

[B36] Maltamo M. Naesset E. Vauhkonen J. (2014). *Forestry applications of airborne laser scanning.* Netherlands: Springer.

[B37] Pont D. (2016). *Assessment of individual trees using aerial laser scanning in New Zealand Radiata Pine forests.* Ph.D thesis, University of Canterbury.

[B38] Pont D. Kimberley M. O. Brownlie R. K. Sabatia C. O. Watt M. S. (2015). Calibrated tree counting on remotely sensed images of planted forests. *Int. J. Remote Sens.* 36 3819–3836. 10.1080/01431161.2015.1054048

[B39] Pont D. Morgenroth J. Watt M. S. (2013). “Tree-based analysis of ALS to estimate tree size and quality,” in *proceeding of the MeMoWood – Measurement Methods and Modelling Approaches for Predicting Desirable Future Wood Properties*, Nancy.

[B40] Pretzsch H. (2010). *Forest dynamics, growth and yield: From measurement to model.* Berlin Heidelberg: Springer-Verlag.

[B41] Pukkala T. Vettenranta J. Kolström T. Miina J. (1994). Productivity of mixed stands of *pinus sylvestris* and *picea abies*. *Scandinavian J. Forest Res.* 9 143–153. 10.1080/02827589409382824

[B42] Ronse C. Heijmans H. J. A. M. (1991). The algebraic basis of mathematical morphology: II. Openings and closings. *CVGIP: Image Understanding* 54 74–97. 10.1016/1049-9660(91)90076-2

[B43] Rouvinen S. Kuuluvainen T. (1997). Structure and asymmetry of tree crowns in relation to local competition in a natural mature scots pine forest. *Can. J. Forest Res.* 27 890–902. 10.1139/x97-012

[B44] Suárez J. C. (2010). *An analysis of the consequences of stand variability in sitka spruce plantations in Britain using a combination of airborne LiDAR analysis and models.* Ph.D thesis, University of Sheffield.

[B45] Suontama M. Low C. B. Stovold G. T. Miller M. A. Fleet K. R. Li Y. (2015). Genetic parameters and genetic gains across three breeding cycles for growth and form traits of *Eucalyptus regnans* in New Zealand. *Tree Genet. Genom.* 11:133 10.1007/s11295-015-0957-8

[B46] Tobler W. (1970). A computer movie simulating urban growth in the Detroit region. *Econom. Geograp.* 1970 234–240. 10.2307/143141

[B47] Tsaftaris S. A. Minervini M. Scharr H. (2016). Machine learning for plant phenotyping needs image processing. *Trends Plant Sci.* 21 989–991. 10.1016/j.tplants.2016.10.002 27810146

[B48] Van Der Pas J. B. Bulman L. Slater-Hayes J. D. (1984). Evaluation of the assessment of Dothistroma needle blight in stands of *Pinus radiata*. *N. Zealand J. Forestry Sci.* 14 3–13.

[B49] Vauhkonen J. Holopainen M. Kankare V. Vastaranta M. Viitala R. (2016). Geometrically explicit description of forest canopy based on 3D triangulations of airborne laser scanning data. *Remote Sens. Environ.* 173 248–257. 10.1016/j.rse.2015.05.009

[B50] Watt M. Bulman L. Palmer D. (2011). The economic cost of Dothistroma needle blight to the New Zealand forest industry. *N. Zealand J. Forestry* 56 20–22.

[B51] White J. C. Wulder M. A. Varhola A. Vastaranta M. Coops N. C. Cook B. D. (2013). *A best practices guide for generating forest inventory attributes from airborne laser scanning data using an area-based approach (Version 2.0).* Victoria: Canadian Wood Fibre Centre.

[B52] Zhen Z. Quackenbush L. Zhang L. (2016). Trends in automatic individual tree crown detection and delineation – Evolution of LiDAR data. *Remote Sens.* 8:333 10.3390/rs8040333

